# Age and cognitive status dependent differences in blood steroid and thyroid hormone concentrations in intact male rats

**DOI:** 10.1186/s12993-019-0161-3

**Published:** 2019-06-30

**Authors:** Jovana Maliković, Daniel Daba Feyissa, Predrag Kalaba, Babak Saber Marouf, Harald Höger, Michaela F. Hartmann, Stefan A. Wudy, Gerhard Schuler, Gert Lubec, Jana Aradska, Volker Korz

**Affiliations:** 1 Department of Pharmaceutical Chemistry, University of Vienna, Vienna, Austria; 2 Core Unit of Biomedical Research, Division of Laboratory Animal Science and Genetics, Medical University of Vienna, Vienna, Austria; 3 Steroid Research & Mass Spectrometry Unit, Peptide Hormone Research Unit, Laboratory for Translational Hormone Analytics in Pediatric Endocrinology, Division of Pediatric Endocrinology & Diabetology, Center of Child and Adolescent Medicine, Justus Liebig University, Giessen, Germany; 4 Veterinary Clinic for Obstetrics, Gynecology and Andrology of Large and Small Animals, Faculty of Veterinary Medicine, Justus Liebig University, Giessen, Germany; 5 Neuroscience Laboratory, Paracelsus Medical University, 5020 Salzburg, Austria

**Keywords:** Aging, Rat, Cognitive decline, Testosterone, Steroid hormones, Thyroid hormones

## Abstract

**Background:**

Age-dependent alterations of hormonal states have been considered to be involved in age related decline of cognitive abilities. Most of the studies in animal models are based on hormonal substitution in adrenal- and/or gonadectomized rodents or infusion of steroid hormones in intact rats. Moreover, the manipulations have been done timely, closely related to test procedures, thus reflecting short-term hormonal mechanisms in the regulation of learning and memory. Here we studied whether more general states of steroid and thyroid hormone profiles, independent from acute experiences, may possibly reflect long-term learning capacity. A large cohort of aged (17–18 months) intact male rats were tested in a spatial hole-board learning task and a subset of inferior and superior learners was included into the analysis. Young male adult rats (16 weeks of age) were also tested. Four to 8 weeks after testing blood plasma samples were taken and hormone concentrations of a variety of steroid hormones were measured by gas chromatography-tandem mass spectrometry or radioimmunoassay (17β-estradiol, thyroid hormones).

**Results:**

Aged good learners were similar to young rats in the behavioral task. Aged poor learners but not good learners showed higher levels of triiodothyronine (T3) as compared to young rats. Aged good learners had higher levels of thyroid stimulating hormone (TSH) than aged poor learning and young rats. Both aged good and poor learners showed significantly reduced levels of testosterone (T), 4-androstenedione (4A), androstanediol-3α,17β (AD), dihydrotestosterone (DHT), 17-hydroxyprogesterone (17OHP), higher levels of progesterone (Prog) and similar levels of 17β-estradiol (E2) as compared to young rats. The learning, but not the memory indices of all rats were significantly and positively correlated with levels of dihydrotestosterone, androstanediol-3α,17β and thyroxine (T4), when the impacts of age and cognitive division were eliminated by partial correlation analyses.

**Conclusion:**

The correlation of hormone concentrations of individuals with individual behavior revealed a possible specific role of these androgen and thyroid hormones in a state of general preparedness to learn.

## Background

Age-dependent decline of cognitive abilities in elderly men has been mostly but not exclusively related to decreased testosterone release [1] and testosterone substitution is used as therapeutic intervention, however controversially discussed [2] and with opposing results [3–5]. However, testosterone is catabolized into several neuroactive and learning and memory affecting steroids such as dihydrotestosterone, which is the most potent androgen receptor agonist, and also in 17β-estradiol by the enzyme aromatase. Estrogen receptors and aromatase are present in brains of male subjects and can exert learning and memory relevant functions [6, 7]. Corticosterone (in rodents) andcortisol (in humans) are stress hormones involved in learning and memory as well as synaptic and neuronal plasticity, when modulations are timely related to the formation of long-term memories. Corticosterone application after acquisition support memory consolidation [8, 9], whereas it impairs memory retrieval when given shortly (30 min) but not hours before a memory retention test [10]. Besides these steroid hormones, thyroid hormones could also be related to cognitive decline in men [11–13]. Due to this variety of neuroactive hormones that can be independently or interactively regulated, it is feasible that the individual profiles of different steroid hormones, rather than the levels of certain hormones determine the cognitive status, especially in aged subjects.

Most of the studies in animal models on steroid hormone related cognitive processes are based on hormonal substitution in adrenal- and/or gonadectomized rodents or infusion of steroid hormones in intact rats. Moreover, the manipulations have been done timely, closely related to test procedures, thus reflecting short-term hormonal mechanisms in the regulation of learning and memory. Here we were interested in a more general state of steroid hormone profiles independently from acute experiences, possibly reflecting long-term learning capacity. For this reason we tested a large cohort of aged (17–18 months) intact male rats in a spatial holeboard learning task and included a subset of inferior and superior learners into the analysis. Young male adult rats (16 weeks of age) were also tested. Four to 8 weeks after testing the blood hormone concentrations of a variety of steroid hormones were measured by gas chromatography-tandem mass spectrometry or radioimmunoassay (17β-estradiol, thyroid hormones).

The study aimed at elucidating age-related differences in hormone levels that may explain differences in cognitive states of aged and young rats and age-independent possible hormonal markers of cognitive abilities.

## Methods

### Subjects

Aged (17–18 months) and young (4 months) male Sprague–Dawley rats, bred and maintained in the Core Unit of Biomedical Research, Division of Laboratory Animal Science and Genetics, Medical University of Vienna were used. Rats were housed in groups of three in standard Makrolon cages filled with autoclaved woodchips (temperature: 22 ± 2 °C; humidity: 55 ± 5%; 12 h artificial light/12 h dark cycle: light on at 7:00 a.m.). Tap water and food (ssniff, R/M-H Ered II, Soest, Germany) was provided ad libitum. The study was carried out according to the guidelines of the Ethics committee, Medical University of Vienna, and were approved by the Federal Ministry of Education, Science and Culture, Austria.

In order to avoid differences in steroid hormone levels due to age related different circadian rhythms blood samples were taken during the light phase at different times, but timely matched between groups. Samples were collected 4–8 weeks after the behavioral test. Animals were anesthetized with Nembutal (40 mg/kg bodyweight) and Heparin (Gilvasan Pharma GmbH, Vienna, Austria) was injected through the tail vein (1000 I.E/kg body weight). After 10 min the animals were decapitated and trunk blood was sampled and centrifuged (9000 rpm for 15 min). Plasma was aliquoted and stored at − 80 °C until measurements.

### Hole-board

The animals were pre-screened for cognitive abilities with the hole-board test. All groups underwent this test procedure before samples were taken. The hole-board board (1 m × 1 m) was manufactured of black plastic surrounded by translucent plexiglass walls. Walls were equipped with proximal spatial cues, and surrounding room structures served as distal cues. Four out of sixteen regularly arranged holes (diameter and depth 7 cm) were baited (dustless precision pellets, 45 mg, Bioserv^®^, Flemington, NJ; USA) with the pattern of baited holes remained the same during the entire test. A second board below the first was provided with scattered food pellets to avoid olfactory orientation. Ten min handling sessions per day for 4 days prior to the experiment made the rats familiar to the experimenter. The following 2 days animals were habituated to the hole-board by free exploration of the maze for 15 min each day with access to food pellets. Controlled food restriction reduced the weight of the rats to reach 85% of their initial body weight. Tap water was given ad libitum. Training consisted of 3 days (five trials on day one, four trials on day 2 and a retention trial at day 3) with an intertrial interval of 20 min for individual rats. Trial duration was 120 s or until all four pellets were eaten. The apparatus was cleaned with 0.1% Incidin between trials in order to remove odor cues of individual rats. Performance of the rats was recorded by a video camera and stored on a computer. The hole visits and removals of pellets were noted for each trial. In order to compare rats with similar levels of motivation, rats with less than 40 hole visits in total over the ten trials were excluded from the analysis.

Reference memory errors were noted as the number of visits to the unbaited holes. Reference memory index (RMI) was calculated using the formula (first + revisits of baited holes)/total visits of all holes. All behavioral training/testing was performed during the light phase of the light–dark cycle. Learning index was calculated as the mean value of reference indices of trials 6–9 at day 2. Memory index is represented by the reference memory index of the retention trial 10 (day 3).

Poor learners were defined when having either learning or memory indices lower than one standard deviation from the mean and good learners when having indices one standard deviation higher than the mean. The rats analyzed in the present study were randomly chosen from good (19 animals) and poor (15 animals) performing animals from a larger cohort of rats (n = 127) with more than 40 hole visits.

### Hormone determinations

Gas chromatography-tandem mass spectrometry (GC–MS/MS) was performed to measure steroid hormones. Briefly, samples were equilibrated with deuterated internal standards, extracted using Extrelut^®^ NT columns and purified using Sephadex LH-20 mini columns. Thereafter, heptafluorobutyrate derivatives were prepared [14]. Gas chromatography was performed on an Optima^®^ 1-MS capillary column (25 m × 0.2 mm I.D., df 0.1 µm, Macherey–Nagel, Düren, Germany) housed in a Thermo Scientific Trace 1310 Gas Chromatograph with a TriPlus RSH Autosampler coupled to a TSQ 8000 triple quadrupole MS (Thermo Scientific, Dreieich, Germany). Helium was used as carrier gas at 1.0 mL/min. The injector temperature was 270 °C and the initial column temperature was set at 80 °C. The steroids of interest eluted at a rate of 3 °C/min until the column temperature reached 242 °C. The following MRM or m/z ratios were measured for the analytes and their corresponding internal standards: m/z 665.1 (668.1) for testosterone (T) (d3-T), m/z 482.2/482.2 (484.3/484.3) for 4-androstenedione (4A) (d2-4A), m/z 455.3/241.3 (458.3/244.4) for androstanediol-3α,17β (AD) (d3-AD), m/z 270.2/121.1 (272.2/123.1) for DHEA (d2-DHEA), m/z 414.1/185.2 (417.2/188.2), for Dihydrotestosterone (DHT) (d3-DHT), m/z 465.2/109.1 (469.1/113.1) for 17-Hydroxyprogesterone (17OHP) (d4-17OHP), m/z 467.2/253.0 (471.3/256.3) for 17-Hydroxypregnenolone (17OH5P) (d7-17OH5P), m/z 465.2/109.1 (467.2/109.1) for 11-deoxycortisol (S) (d2-S),705.1/355.1 (712.1/359.2) for corticosterone (B) (d8-B), and m/z 510.2/495.2 (/518.3/503.4) for progesterone (Prog) (d9-Prog).

Concentrations of 17β-estradiol (E2) were measured by a sequential radioimmunoassay applying tritiated tracer and an antiserum generated against E2-6-carboxymethyl oxime—BSA after extraction of the samples with toluene [15].

Total T3 and total T4 were measured by a radioimmunoassays (Beckman Coulter, Krefeld, Germany). TSH was measured using an immunoradiometric assay (Beckman Coulter, Krefeld, Germany).

The hormones that were quantitatively identified are listed in Table 1. For three hormones (DHEA, 17OH5P and S) all values of all groups were below the limit of detection, therefore these hormones were excluded from the analysis. Values below the detection limit were set to half of the limit value and included in the analysis. Number of measurements below the detection limit was: 4A: 8 aged good, 7 aged poor, 4 young; AD: 1 aged good, 5 aged poor, 2 young; DHT: 4 aged good, 2 aged poor, 17OHP: 5 aged good, 8 aged poor, 2 young; Prog: 8 young; TSH: 1 aged good, 8 aged poor, 9 young.

**Table 1:** List of measured hormones with abbreviations, chemical and trivial names and detection limits

Hormone	Chemical name	Trivial name	Detection limit
T	4-Androsten-17β-ol-3-on	Testosterone	0.1 ng/mL
4A	4-Androsten-3,17-dion	4-Androstenedione	0.1 ng/mL
AD	5a-Androstan-3α,17β-diol	Androstanediol-3α,17β	0.1 ng/mL
DHEA	5-Androstene-3β-ol-17-one	Dehydroepiandrosterone	0.5 ng/mL
DHT	5a-Androstan-17β-ol-3-on	Dihydrotestosterone	0.025 ng/mL
17OHP	4-Pregnen-17α-ol-3,20-dion	17-Hydroxyprogesterone	0.1 ng/mL
17OH5P	5-Pregnene-3β,17α-diol-20-one	17-Hydroxypregnenolone	0.1 ng/mL
S	4-Pregnene-17α,21-diol-3,20-dione	11-Deoxycortisol	0.1 ng/mL
Prog	4-Pregnen-3,20-dion	Progesterone	0.625 ng/mL
B	4-Pregnen-11β,21-diol-3,20-dion	Corticosterone	5 ng/mL
E2	1,3,5(10)-Estratrien-3-ol-17-one	17β-Estradiol	2 pg/mL
T3	3,3′,5-Triiod-l-thyronin	Triiodothyronine	0,75 nmol/L
T4	3,3′,5,5′-Tetraiod-l-thyronin	Thyroxine	26 nmol/L
TSH	5-Oxo-l-prolyl-l-histidyl-l-prolinamide	Thyroid-stimulating-hormone	0.15 mLU/L

### Statistics

Group differences between hormone levels and behavior were analyzed by two-way multivariate general linear model (ANOVA) with hormone levels and age/performance as factors and subsequent Bonferroni post hoc tests. Correlations between individual levels of hormones and learning and memory indices were done by partial correlation analyses with age and predetermined cognitive status as controlling variables. Sample sizes: aged good (n = 10), aged poor (n = 10), young (n = 10). Analyses were done by using SPSS statistics program (V. 20).

## Results

### Group differences in learning and memory

**Fig. 1:**
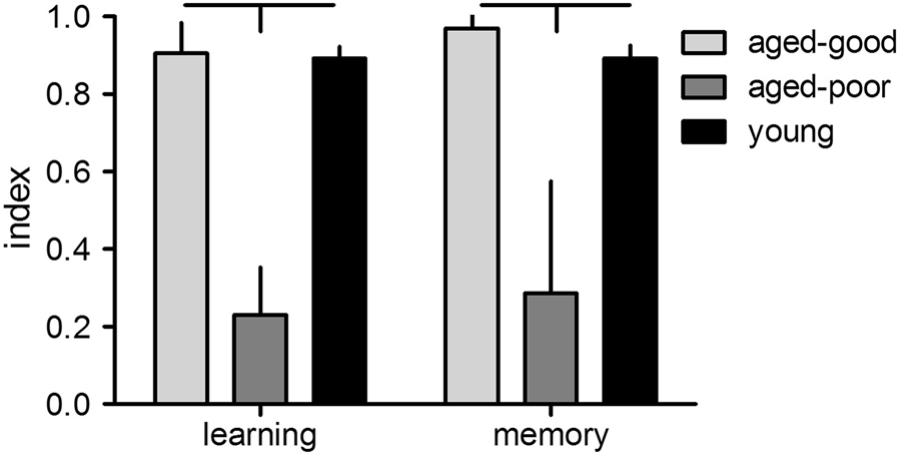
Learning (mean reference memory indices of trial 6–9 at training day 2) and memory (reference memory index of trial 10 at day 3) of aged good and poor learners as well as young rats (n = 10, each). Horizontal and vertical bars indicate statistically significant differences between groups. Given are the means with standard deviation

The results are summarized in Fig. 1. We could determine an overall difference in behavioral performance between groups both in learning (F_2,27_ = 149.4, p < 0.0001) and memory (F_2,27_ = 40.6, p < 0.0001) indices. Aged good-learning rats show significantly better performance in learning and memory as compared to aged poor-learning rats (p < 0.001, each) but not as compared to young rats (p = 1.0, each). Young rats performed better as compared to aged poor-learning rats (p < 0.001, each).

### Age-dependent differences in hormone levels

**Fig. 2:**
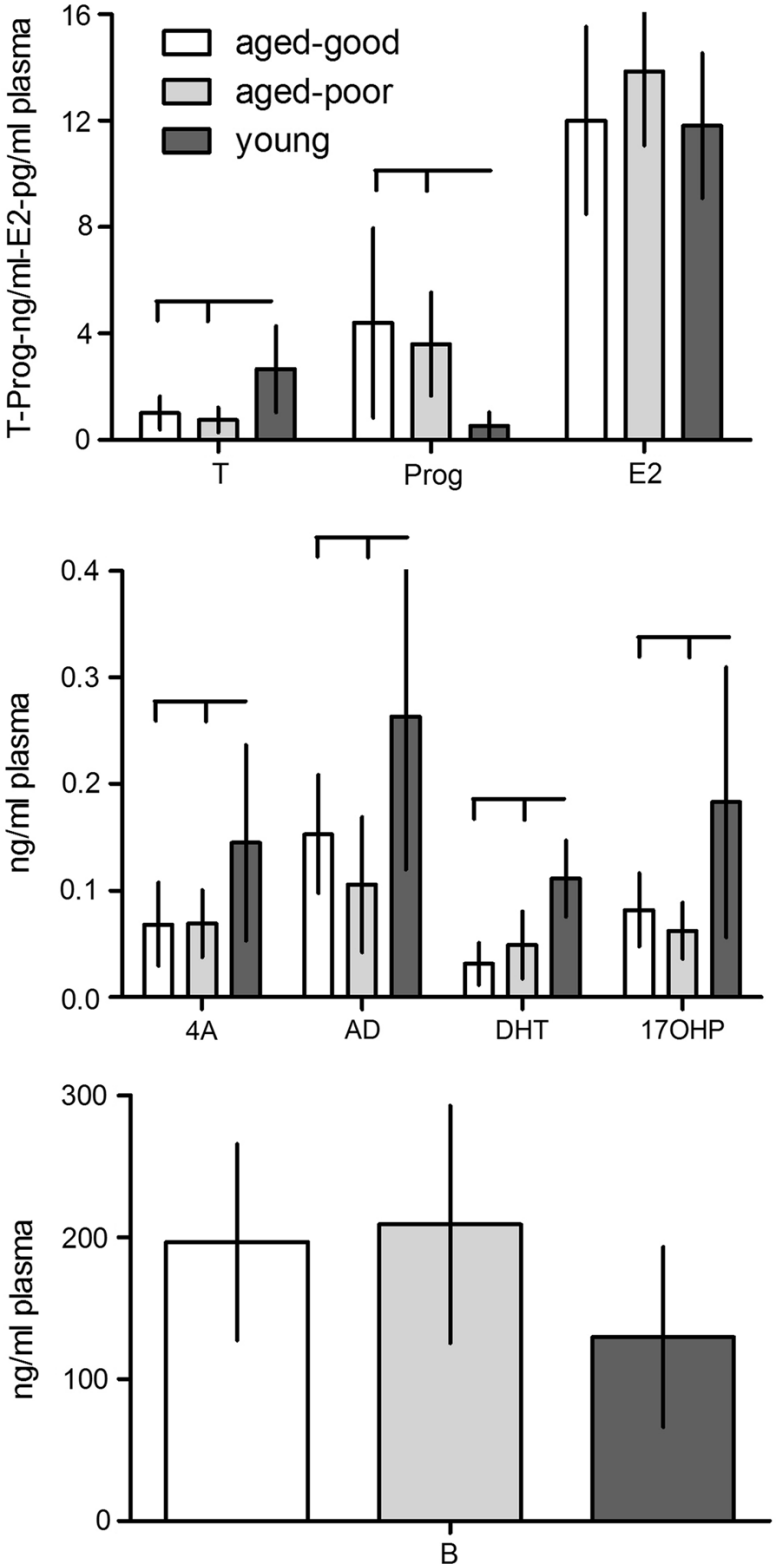
Plasma steroid hormone concentrations of aged good and poor learners and young rats (n = 10, each). T, testosterone; Prog, progesterone; E2, 17β-estradiol; 4A, 4-androstenedione; AD, androstanediol-3α,17β; DHT, dihydrotestosterone; 17OHP, 17-hydroxyprogesterone; B, corticosterone. Horizontal and vertical bars indicate statistically significant differences between groups. Given are the means with standard deviation

**Fig. 3:**
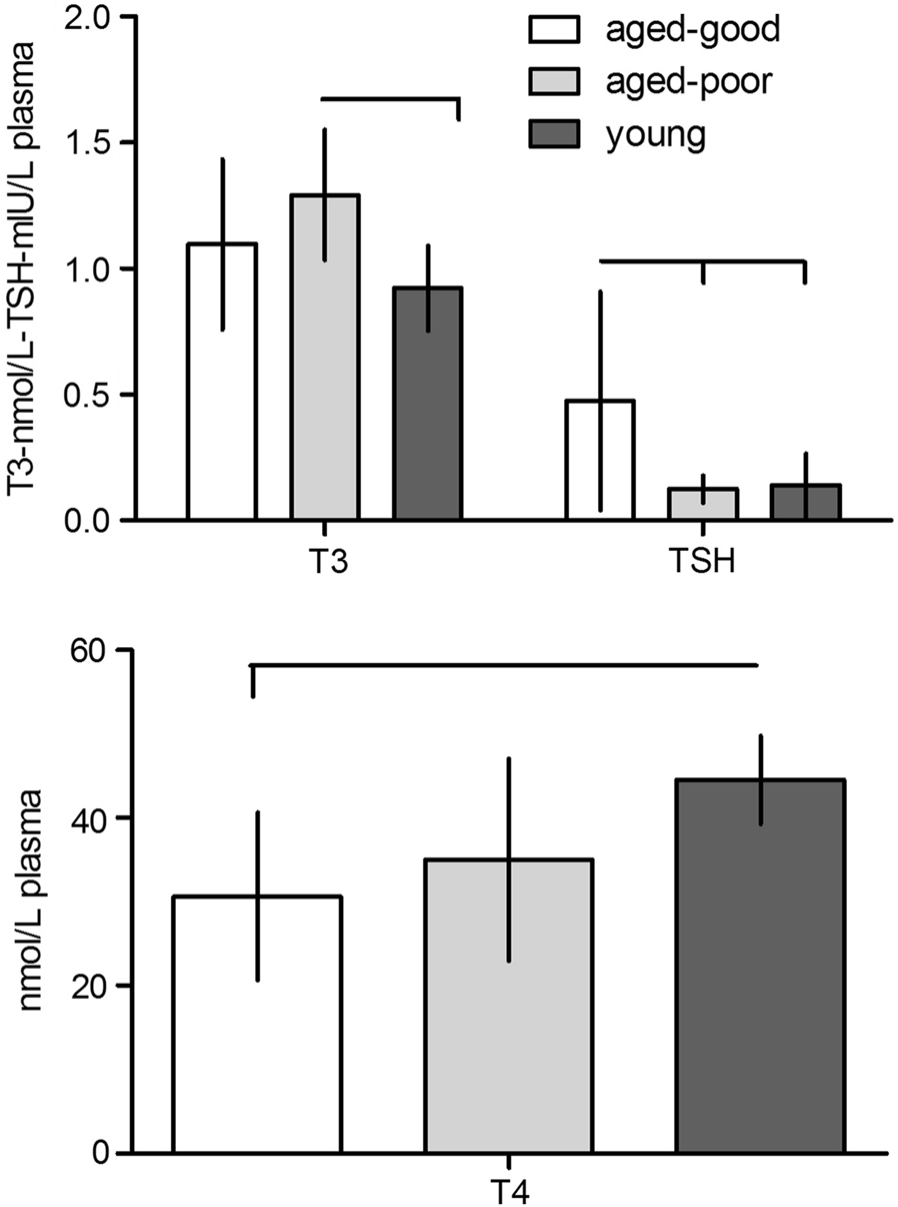
Plasma concentrations of thyroid hormones of aged good and poor learners and young rats (n = 10, each). T3, triiodothyronine; T4, thyroxine; TSH, thyroid-stimulating-hormone. Horizontal and vertical bars indicate statistically significant differences between groups. Given are the means with standard deviation

The results are given in Figs. 2, 3. Overall significant effects between groups could be detected. Testosterone (T): F_2,27_ = 9.92, p = 0.001; 4-Androstenedione (4A): F_2,27_ = 5.34, p = 0.011; Androstanediol-3α,17β (AD): F_2,27_ = 7.11, p = 0.003; Dihydrotestosterone (DHT): F_2,27_ = 19.95, p < 0.001; 17-Hydroxyprogesterone (17OHP): F_2,27_ = 6.97, p = 0.004; Progesterone (Prog): F_2,27_ = 7.56, p = 0.002; Corticosterone (B): F_2,27_ = 3.46, p = 0.046; Triiodothyronine (T3): F_2,27_ = 4.89, p = 0.015; Thyroxine (T4): F_2,27_ = 5.54, p = 0.010; and Thyroid-stimulating- hormone (TSH): F_2,27_ = 5.65, p = 0.009, but not 17β-estradiol (E2): F_2,27_ = 1.37, p = 0.271.

Post-hoc tests revealed higher levels of young vs. aged good learners or aged poor learners for testosterone (p = 0.004 and p = 0.001; respectively), 4-Androstenedione (p = 0.022 and p = 0.023; respectively); Dihydrotestosterone (p < 0.001 and p < 0.001; respectively); 17-Hydroxyprogesterone (p = 0.019 and p = 0.005; respectively). Young rats showed lower levels of Progesterone as compared to aged good and aged poor rats (p = 0.003 and p = 0.021; respectively). No significant differences for these hormones could be determined between aged good learners and aged poor learning rats (p > 0.05 each). Androstanediol-3α,17β levels where higher in young vs. aged poor learners (p = 0.003) and aged good learning rats (p = 0.048), with no differences between aged good and aged poor learning rats (0.521). Triiodothyronine titers were higher in aged poor learners as compared to young (p = 0.013), but there was no difference between aged poor and aged good learners (p = 0.328) or aged good learners and young rats (p = 0.459). Thyroxine levels were lower in aged good learners as compared to young (p = 0.009), but no difference could be found when compared to aged poor learners (p = 0.944) and no difference between the two latter was observed (p = 0.102). Thyroid-stimulating-hormone levels were elevated in aged good as compared to aged poor learners (p = 0.018) and young (p = 0.025) rats, but no difference could be determined between aged poor learners and young rats (p = 1). We could not detect significant differences between single groups for Corticosterone (young vs. aged good p = 0.147; young vs. aged poor p = 0.064; aged good *vs*. aged poor p = 1)) and 17β-estradiol (young *vs*. aged good p = 1; young *vs*. aged poor p = 0.434; aged good *vs*. aged poor p = 0.559).

### Correlations

The results of partial correlations with learning and memory indices are summarized in Table 2. Two control variables were used, one with predetermined cognitive status dividing the sample into good (aged good and young) and poor (aged poor) learners and the other with age, dividing the sample into aged (aged good and aged poor) and young rats. Partial correlations allows to avoid misleading results if confounding variables numerically related to both variables of interest. Confounding variables here are the predetermined separation of good and poor learners and the other is the difference in age. Thus, correlations between cognitive states and hormones independently of the predetermination can be calculated. Similarly with the second control variable correlations independently of age can be determined. Significant correlations of Androstanediol-3α,17β, dihydrotestosterone and thyroxine levels could be found with learning but not with memory indices.

**Table 2:** Partial correlations between hormone levels and learning and memory indices (n = 10 for each group, n = 30 in total)

	T	4A	AD	DHT	17OHP	Prog	E2	B
Learning index								
Coeff.	0.326	0.267	*0.398*	*0.409*	0.173	− 0.092	0.090	− 0.043
p	0.090	0.169	*0.036*	*0.031*	0.379	0.642	0.650	0.830
Memory index								
Coeff.	− 0.168	− 0.215	− 0.272	− 0.161	− 0.199	0.205	0.207	0.332
p	0.394	0.271	0.162	0.414	0.311	0.295	0.291	0.085

	T3	T4	TSH					
Learning index								
Coeff.	0.058	*0.460*	0.191					
p	0.770	*0.014*	0.331					
Memory index								
Coeff.	− 0.311	− 0.100	0.051					
p	0.108	0.613	0.795					

Given are the coefficients (Coeff.) and related p-values (p). Significant correlations are indicated in italics. The division into good and poor learners and the different age were addressed by appropriate control variables

In Table 3 the partial correlations between individual hormone levels are presented. Whereas most of the androgenic hormone levels are positively intercorrelated and correlated with 17-Hydroxyprogesterone, the levels of T3 and T4 are positively intercorrelated but not correlated with TSH, which levels are inversely correlated with progesterone. In addition we found a positive correlation between progesterone and corticosterone levels.

**Table 3:** Partial correlations between hormone levels (n = 10 for each group, n = 30 in total)

	4A	AD	DHT	17OHP	Prog	E2	B	T3	T4	TSH
T										
Coeff.	*0.879*	*0.936*	*0.807*	*0.871*	0.020	0.155	− 0.085	0.179	0.142	− 0.039
p	*0.000*	*0.000*	*0.000*	*0.000*	0.921	0.432	0.669	0.362	0.471	0.843
4A										
Coeff.		*0.796*	*0.677*	*0.842*	0.041	0.231	− 0.113	0.301	0.074	− 0.074
p		*0.000*	*0.000*	*0.000*	0.837	0.238	0.566	0.120	0.707	0.706
AD										
Coeff.			*0.763*	*0.796*	− 0.002	− 0.013	− 0.131	0.158	0.039	− 0.053
p			*0.000*	*0.000*	0.991	0.947	0.506	0.422	0.845	0.788
DHT										
Coeff.				*0.694*	0.095	0.019	0.124	0.322	0.364	− 0.111
p				*0.000*	0.631	0.922	0.531	0.094	0.057	0.575
17OHP										
Coeff.					0.084	0.127	− 0.133	0.386	0.185	− 0.004
p					0.671	0.520	0.499	0.042	0.346	0.984
Prog										
Coeff.						− 0.201	*0.565*	0.276	0.110	− *0.413*
p						0.305	*0.002*	0.156	0.578	*0.029*
E2										
Coeff.							− 0.088	− 0.245	− 0.126	0.253
p							0.656	0.209	0.524	0.194
B										
Coeff.								− 0.028	0.116	− 0.144
p								0.888	0.555	0.464
T3										
Coeff.									*0.437*	− 0.078
p									*0.020*	0.692
T4										
Coeff.										0.145
p										0.463

Given are the coefficients (Coeff.) and related p-values (p). Significant correlations are indicated in italics. The division into good and poor learners and the different age were addressed by appropriate control variables

## Discussion

In order to reveal hormone related long-term learning capacities independent from actual learning experiences blood plasma was sampled 4–8 weeks after a hole-board test, which was conducted to test for individual cognitive capacities. Although we cannot completely rule out that during this time period age related changes in hormone status may take place, this is unlikely. Tang [16] and Waner and Nyska [17] found only slight differences of thyroid hormones in male rats at ages comparable with the present study and even at larger differences in age (12–18 months). Similarly, testosterone is slightly affected during the age period considered in the present study [18]. We found age dependent differences between steroid and thyroid hormones independently from cognitive status and also cognitive status dependent different results between aged and young animals. Namely the levels of Androstanediol-3α,17β are significantly reduced in aged poor and significantly (but close to the border of significance) in aged good learning rats as compared to young rats. The levels of TSH are significantly enhanced in aged good as compared to aged poor learners and young rats. Further, age independent positive correlations with learning but not memory indices could be detected for AD, DHT and T4.

AD, a metabolite of dihydrotestosterone (DHT), is a neuro-steroid binding to the gamma-aminobutyric acid (GABA_A_) receptor as a positive allosteric modulator increasing GABA responses up to 50% in hippocampal CA1 pyramidal cells effectively regulating neuronal excitability [19]. AD has been shown to interact with cytoplasmic estrogen receptors in the brain, although to a much lesser extent than its 3β isomer [20]. AD has been described to have rewarding and anxiolytic effects [21, 22]. AD has also effects on learning, conditioned place preference was enhanced by sub-chronic application of AD to a higher extent than by administration of DHT or testosterone [23]. Gestational stress in male rats produced behavioral inhibition in adult life, correlated with increased levels of corticosterone and reduced levels of DHT and AD [24]. AD but not testosterone application reinstates age-related impaired cognitive performance in aged male rats and enhanced performance in spatial learning (water maze) irrespective of age [25]. However, intrahippocampal infusion of AD impaired water maze performance in adult male rats and decreased transcription levels of protein kinase A (PKA) [26]. PKA is a critical mediator of spatial learning and memory and synaptic plasticity [27–30].

DHT is catabolized from testosterone by the enzyme 5α reductase and is a considerably more potent agonist of the androgen receptor than testosterone in peripheral [31] and brain tissue [32]. Although, testosterone levels decline with age, there is little evidence that testosterone substitution rescues spatial cognitive abilities in aged rats, however it affects memory in young rats [33], whereas in humans most, but not all, of the studies report enhancement of cognition after testosterone replacement in healthy aged men [1]. DHT is metabolized into AD by the enzyme 3α-HSD (3α-hydroxysteroid dehydrogenase). The partial correlation analysis in the present study support the view that not testosterone itself, but the metabolites DHT and AD are involved in the determination of learning capacities in an age-independent manner. The synthesis of AD may be in part independent of the availability of DHT. Although there are significantly decreased levels of DHT and AD in both aged groups compared to young rats, the significance is weak in aged good learners and the levels of AD are slightly higher as in the aged poor learners. This may be regulated by different levels or activity of 3α-HSD in aged good vs. aged poor rats. 3α-HSD hippocampal mRNA levels decrease with age in rats [34], which can be attenuated by environmental enrichment experience. Intrahippocampal application of indomethacin, a 3α-HSD inhibitor, impairs leaning but not memory consolidation in a spatial water maze task [35]. However, the present group specific AD data only allow limited interpretations but should be proved in further studies.

The correlation data suggest these neuroactive steroids may represent a age independent marker for a consistently elevated learning capacity, whereas the formation of a long-term memory is probably regulated by short-term hormonal mechanisms closely related to the memory acquisition and consolidation phases and becomes independent from hormonal states long after consolidation. Scheinert et al. [36] found correlations over young, middle aged and aged rats of some cytokines, chemokines, corticosterone and adrenocorticotropic hormone (ACTH) from samples taken 2 weeks after water maze training with learning and memory indices and differences in concentrations in serum, hippocampus and cortex in dependence of the cognitive status of the rats. Thus, the cognitive status is reflected in some physiological parameters over a long time. Similarly, Issa et al. [37] found that HPA axis dysfunction in aged rats is associated with spatial memory impairments and not merely a function of age. Age dependent and independent hypothalamus–pituitary–adrenal (HPA)-axis adjustments to determine learning abilities were also found by Meijer et al. [38]. Aged inferior but not superior male rat learners show a positive correlation of arginine vasopressin mRNA in the parvocellular nucleus of the hypothalamus with basal blood corticosterone levels, suggesting impaired glucocorticoid sensitivity. Here we did not find a correlation of corticosterone with learning and memory indices. However, corticosterone can be synthetized independently of the HPA axis activity from progesterone in rat testis [39, 40]. Thus, the levels of corticosterone, especially in aged rats, may be in part based on the highly available progesterone, which is also suggested by the positive correlation of progesterone and corticosterone at individual levels. This correlation can be also found, when young rats are excluded. Enhanced progesterone levels in aged male rats as shown here have been reported previously [41, 42]. The increased progesterone levels contribute to the suppression of gonadotropins and impaired reproductive functions in aged males [43].

Literature results related to effects of aging on thyroid hormones are controversial, age dependent decline of T3 and T4 in blood of male rats [16, 17, 44] or no differences [45] have been reported. Diminished levels of T4 but not T3 have also been found [46]. While some found decreased levels of TSH over age [45] others found no difference [44, 46, 47] or increased levels of TSH in aged males [48].

Thyroid hormones especially improve hippocampus-dependent learning and memory and hippocampal synaptic plasticity [49–51] as well as hippocampal neurogenesis [52]. Thyroxine treatment improves spatial learning in a water maze probably by induced increased cholinergic activity [53] and rescues spatial cognitive deficits and dentate gyrus electrical activity in a rat model of Alzheimer’s disease [54]. T3 and T4 reduce GABA-evoked and spontaneous inhibitory synaptic currents up to 50%, whereas T4 in contrast to T3 was ineffective in decreasing extra-synaptic GABA currents [55]. Thus, possibly T3 and T4 in conjunction with AD may effectively regulate hippocampal and probably extrahippocampal network activities to facilitate cognitive functionality during spatial learning.

TSH levels have been found to be positively related to episodic memory in aged humans (75–96 years) independently from the actual age [11]. However, *van* Boxtel et al. [56] found a weak inverse relation of TSH and cognition in aged individuals, which was dependent on mood status. TSH shows potent neuroprotective properties. TSH injections protected against electroconvulsive disruption of memory retrieval. This effect was independent from the TSH induced levels of plasma T3 and T4 [57]. Early thyroxine treatment improves spatial learning and memory and enlarges intra- and infrapyramidal mossy fiber projections in the hippocampus. Individual sizes of these projections were positively correlated with radial maze performance [58].

Thus, TSH in the present study may have cognitive enhancing functions in aged but not young rats independently of T3 and T4. Metanalytic studies in humans revealed an association of TSH with poor cognitive performance in younger but better performance in older subjects on a variety of tests, whereas thyroxine levels show such a relation only for a single test [12]. Low TSH levels could be related to a progression of cognitive impairment to dementia [13].

The present study, by analyzing a large number of hormones in the same individuals, can point to some possible underlying mechanisms of hormonal learning and memory modulations in an age dependent and independent manner. Especially the role of TSH as a potential biomarker for cognitive decline in elderly but not young subjects, and the applicability of dihydrotestosterone, androstanediol-3α,17β and thyroxine as age independent biomarkers for hormone related alterations of cognitive abilities should be proved in further studies. This studies should also include a measure of these critical hormones before and after behavioral testing, which would be possible by the decreased amount of plasma that is needed for the analysis. Further measurements in brain tissue are of interest.

## Conclusion

The major outcome of the study is that aged good learners were similar to young rats. Aged poor learners, but not good learners showed higher levels of triiodothyronine as compared to young rats. Aged good learners had higher levels of thyroid stimulating hormone than aged poor learning and young rats. Both, aged good and poor learners showed significantly reduced levels of testosterone, 4-androstenedione, androstanediol-3α,17β, dihydrotestosterone, 17-hydroxyprogesterone, higher levels of progesterone and similar levels of 17β-estradiol as compared to young rats. The learning, but not the memory indices of all rats were significantly and positively correlated with levels of dihydrotestosterone, androstanediol-3α,17β and thyroxine, when the impacts of age and cognitive division were eliminated by partial correlation analyses. Analysis of individual hormonal profiles rather than group comparisons revealed a possible specific role of these androgen and thyroid hormones in a state of general preparedness to learn.

## Abbreviations

RMI: reference memory index
T: testosterone
A: 4-androstenedione
AD: androstanediol
DHT: dihydrotestosterone
17OHP: 17-OH-progesterone
17OH5P: 17-OH-pregnenolone
S: 11-deoxycortisol
B: corticosterone
Prog: progesterone
E2: 17β-estradiol
GABA: gamma-aminobutyric acid
PKA: protein kinase A
3α-HSD: 3α-hydroxysterioid dehydrogenase

## Availability of data and materials

The datasets used and/or analyzed during the current study are available from the corresponding author on reasonable request.
